# Intra-Rater Reliability and Construct Validity of Hand-Held Dynamometry to Evaluate the Hip Adductor Squeeze Test in Elite Youth Football Players

**DOI:** 10.3390/sports14020053

**Published:** 2026-02-03

**Authors:** Alexandros Stefanakis, Shane Gore, Christopher Hicks, Michael Mansfield, Matthew Willett

**Affiliations:** 1 School of Sport, Exercise and Rehabilitation Sciences, University of Birmingham, Birmingham B15 2TT, UK; a_stefanakis14@yahoo.gr (A.S.); m.mansfield@bham.ac.uk (M.M.); 2 Millwall Football Club, The Den, Zampa Road, London SE16 3LN, UK; 3 UK Sports Institute, British Canoeing, Lee Valley White Water Centre, Station Road, Waltham Cross, London EN1 1AB, UK; 4 Centre of Precision Rehabilitation for Spinal Pain, University of Birmingham, Birmingham B15 2TT, UK

**Keywords:** dynamometry test, hip adductor squeeze test, football, reliability, construct validity

## Abstract

Hip and groin injuries are common in elite football, and reduced isometric adductor strength has been identified as a key risk factor. Therefore, reliable and valid field-based methods for assessing hip adduction strength are essential for effective monitoring and injury prevention. This study aimed to evaluate the intra-rater reliability and construct validity of a hand-held dynamometer (HHD) compared with the ForceFrame (FFS) during the adductor squeeze test in elite youth football players. Thirty-eight male academy athletes completed two testing sessions four weeks apart, performing maximal isometric adductor squeezes using both devices. Relative reliability was assessed using intraclass correlation coefficients (ICC), and construct validity was evaluated using paired *t*-tests, Bland–Altman analysis, and linear regression. The HHD demonstrated excellent intra-rater reliability (ICC = 0.90, 95% CI: 0.82–0.95) and the FFS showed good reliability (ICC = 0.85, 95% CI: 0.71–0.92). Paired *t*-tests revealed no significant differences between devices, and regression analysis confirmed no proportional bias, indicating strong agreement and construct validity. These findings demonstrate that the HHD provides valid and reliable measurements of isometric hip adduction strength and may serve as a practical, portable, and cost-effective alternative to fixed dynamometry for field-based strength assessment, rehabilitation monitoring, and injury-prevention screening in elite football environments.

## 1. Introduction

Hip and groin injuries are prevalent in elite youth football, with an incidence of 25% and a re-injury rate of 75%, respectively [1]. Hip and groin injuries account for approximately 12–16% of all time-loss injuries in professional footballers [2,3], and recent estimates place their economic burden at EUR94,000 per 1000 h of player exposure in elite European leagues [4].

Reduced isometric adductor strength increases the risk of sustaining a hip and groin injury [5,6,7,8]. The gold standard method for measuring isometric hip adduction strength in elite football is the short-lever hip adductor squeeze test (AST) using a fixed dynamometry system. While isokinetic dynamometry is often considered the gold standard for lab-based strength testing, these machines are not widely accessible in professional football due to their high costs, lack of portability, and the complex setup and calibration required prior to testing. By contrast, the ForceFrame System (FFS) [9] offers a practical alternative to isokinetic dynamometry to assess athlete adductor strength. The FFS is commonly used in elite football environments and has demonstrated excellent reliability when evaluating the AST (Intraclass Correlation Coefficients [ICCs] 0.90–0.95) [9,10]. However, the FFS is less useful in on-field settings compared to more portable alternatives. Hand-held dynamometers (HHDs) use the same testing position as the FFS to evaluate the AST but are less expensive and easier to use to assess isometric hip adduction strength [9]. Although HHDs are becoming increasingly adopted in clinical and sports medicine settings [11], the proportion of professionals using them remains undocumented, highlighting a need for clearer guidance regarding optimal dynamometry selection in elite sport settings. Nevertheless, previous studies have reported inconsistent intra-rater reliability (ICCs range 0.40–0.95) [7,8,12,13], with the variability attributed to a lack of external fixation, non-standardised protocols, and strength differentials between subjects and testers [9,10,14].

Construct validity refers to whether a measurement tool (e.g., HHD) accurately assesses the theoretical domain it is targeting (e.g., isometric hip adduction strength) [14]. A tool must demonstrate consistent intra-rater reliability under the same testing conditions to be considered valid for assessing a construct [15] to be able to confidently justify its use in sports settings. This study aimed to determine the intra-rater reliability and construct validity (using the FFS as the reference standard) of the HHD to measure isometric hip adduction strength.

## 2. Materials and Methods

### 2.1. Study Design

This was a repeated measures study reported in line with the Guidelines for Reporting Reliability and Agreement Studies [15] Supplementary File S1, Table S1. Ethical approval was obtained from the University of Birmingham’s School of Sport, Exercise and Rehabilitation Sciences (Reference: MCR2122_04). All participants and/or their guardians provided written informed consent prior to data collection.

### 2.2. Participants

Elite youth male football players aged 15–21 years were recruited from a professional football club in East London, UK. All players were part of the club’s Professional Development Phase under the Elite Player Performance Plan and trained 5–6 days per week while competing in national elite level competitions [16]. These players compete in the highest-tier national youth leagues, reflecting a high-performance training and competitive environment. To ensure transparency and minimise coercion, participants were approached by their allocated academy physiotherapist (CH), who explained the purpose and procedures of the study and provided written participant information and consent forms. Testing was then conducted by a separate practitioner (SH) who had no involvement in player selection, training, or match-day decisions. All measurements were recorded using the VALD FFS, with the raw data stored securely on the VALD Hub cloud platform. The primary researcher (AS) accessed and extracted anonymised data for statistical analysis.

### 2.3. Inclusion and Exclusion Criteria

Players had to be classified as “fully fit” to participate. In professional sports settings, an athlete’s fitness to train and play is commonly evaluated using the Acute to Chronic Workload Ratio (ACWR) [17]. The ACWR compares an athlete’s recent training (i.e., the past week) to their longer-term performance, and a ratio of greater than 1.5 potentially increases the risk of injury [17]. Fully fit players were defined as those who had undergone medical examination by the club’s medical staff and had an ACWR of between 0.8 and 1.3 [17]. Players were excluded if they sustained an injury between testing sessions, were unable to complete training or match play, or had an ACWR below 0.8 due to reduced training availability. Additionally, pain levels were monitored during all test procedures using a Visual Analogue Scale ranging from 0 to 10. In line with previous research [18], any player who reported pain equal to or greater than 2/10 during the test was excluded to avoid influence from symptomatic groin discomfort. Participants’ eligibility criteria are explained in Table 1.

### 2.4. Researchers and Familiarisation Procedure

All data collection procedures were performed by the following three researchers: AS: a chartered physiotherapist with seven years of experience in sports medicine; SG: a chartered physiotherapist with three years of sports medicine experience; and CH: a sports rehabilitator with four years of clinical experience. Each researcher had extensive experience with musculoskeletal strength testing using both the FFS and HHD. Prior to formal data collection, a familiarisation session was conducted with nine non-participating academy players to standardise testing procedures and ensure consistency in protocol application.

### 2.5. Testing Location and Scheduling

Testing was conducted in the medical rooms of the academy and first-team training facilities between September and October 2021. U16 and U18 squads were tested at the academy site, while U21 players were tested at the senior training ground. To minimise group contamination, different testing times were used: U18s in the morning, U16s in the evening, and U21s on separate days. Testing took place two days after the players’ last match (matchday + 2) and before their training session to ensure standardisation, in accordance with the club’s weekly macrocycle [19].

### 2.6. Equipment and Testing Protocol

Two devices were used for the AST:

- Hand-Held Dynamometer: MicroFET^®^2 (Hoggan Scientific, Salt Lake City, UT, USA)
- ForceFrame System: VALD Performance (Brisbane, Australia)

**Figure 1:**
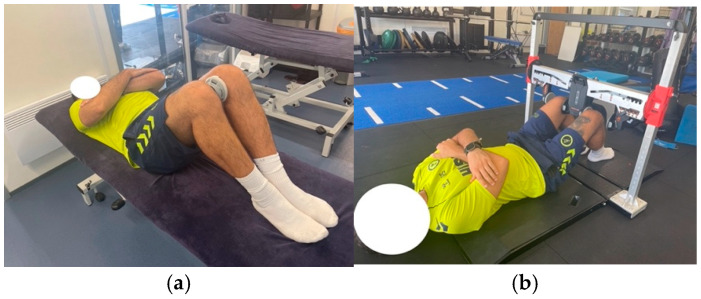
Hip Adductor Squeeze Test (AST): (**a**) position using HHD and (**b**) position using FFS.

During testing, players were guided through the full warm-up and test protocol to ensure consistency and comfort with the procedures. Each full testing session lasted approximately 10 min per player. The same testing protocol was repeated after a four-week interval to assess intra-rater reliability. Each participant attended two test sessions, during which they completed the short-lever adductor squeeze test using both devices. Testing followed the protocol outlined in Light et al. (2019) [10]: The HHD was placed on the medial aspect of the right leg, aligned with the bulk of the vastus medialis oblique, with the player lay supine with hips at 45° flexion and knees at 90°, feet flat on the plinth, and arms crossed over the chest (Figure 1a). The FFS used a bilateral force plate positioned medially, and data were collected on the right limb for consistency (Figure 1b).

Each player completed two submaximal familiarisation contractions, followed by three maximal voluntary contractions (MVCs), each lasting 5 s with 5 s rest intervals. Standardised verbal instructions were used: “Go ahead—push—push—push—relax” [20]. To reduce muscle fatigue, HHD testing was performed first, followed by a two-minute rest, and then FFS testing. All devices were calibrated prior to testing in accordance with manufacturer guidelines.

### 2.7. Statistical Analysis

All statistical analyses were performed using SPSS Statistics Version 24 (IBM Corp., Armonk, NY, USA). Descriptive statistics were calculated for participant characteristics, including age, height, body mass, and footedness. The mean of three MVCs was used for all strength measures. Data normality was assessed using the Shapiro–Wilk test.

#### 2.7.1. Intra-Rater Reliability

Intra-rater reliability was assessed by repeating the strength assessments on two separate occasions, four weeks apart, using the same assessor. Relative reliability was determined using ICCs, classified as excellent (≥0.80), good (0.60–0.79), or poor (<0.60) [21].

Absolute reliability was assessed using the following metrics:

- Standard Error of Measurement (SEM): SEM = SD × √(1 − ICC);
- Relative SEM (SEM%): SEM/mean score × 100;
- Minimal Detectable Change (MDC): MDC = SEM × 1.96 × √2;
- Relative MDC (MDC%): MDC/mean score × 100;
- Coefficient of Variation (CV%): (SD/mean) × 100.

Agreement between both testing scores was further examined using Bland–Altman plots and 95% limits of agreement (LOA).

#### 2.7.2. Construct Validity

Construct validity of the HHD was assessed by comparing its outputs to those of the FFS, which served as the reference standard. Paired *t*-tests were used to assess differences in mean values between devices. Bland–Altman plots and linear regression analysis were used to assess agreement and proportional bias between measurement systems. Statistical significance was set at *p* < 0.05.

## 3. Results

### 3.1. Descriptive Data

A total of 38 male academy football players completed strength assessments on two separate occasions, four weeks apart, with no injuries or adverse events reported between sessions. The sample consisted of 18 Under-16, 12 Under-18, and 8 Under-21 players. Participants’ mean (±SD) age was 16.4 ± 1.5 years, height 181.7 ± 6.3 cm, and body mass 74.4 ± 7.1 kg. Thirty-two players were right-foot dominant, and six were left-foot dominant.

All strength variables met the assumption of normality according to the Shapiro–Wilk test (*p* > 0.05), supporting the use of parametric methods for reliability and agreement analyses. Mean adductor squeeze force values obtained using the HHD were 249.9 ± 58.9 N during the first assessment and 262.2 ± 58.8 N during the second assessment, while corresponding values recorded with the FFS were 373.9 ± 73.6 N and 382.1 ± 76.5 N, respectively. Descriptive characteristics of the player cohort are presented in Supplementary File S1, Table S2, and descriptive force outputs for each device across testing occasions are provided in Supplementary File S1, Table S3, respectively.

### 3.2. Intra-Rater Reliability

The HHD and the FFS demonstrated high consistency between sessions on both occasions. The HHD showed excellent intra-rater reliability with an ICC of 0.90 (95% CI: 0.82–0.95), while the FFS showed good reliability with an ICC of 0.85 (95% CI: 0.71–0.92). The SEM was 12.3 N for the HHD and 21.5 N for the FFS, corresponding to relative SEM values of 4.8% and 5.6%, respectively. The MDC was 34.2 N for the HHD and 59.6 N for the FFS, equivalent to MDC% values of 13.6% and 15.5%. The CV% was 5.5% for the HHD and 6.0% for the FFS. All values are demonstrated in Table 2. Bland–Altman plots indicated that all data points lay within the 95% (LOA), with narrow values (HHD: 11.75; FFS: 14.96), confirming good agreement and minimal measurement error across repeated assessments. Collectively, these results indicate strong relative and absolute intra-rater reliability for both devices in measuring isometric hip adduction strength in elite youth football players.

### 3.3. Construct Validity

**Figure 2:**
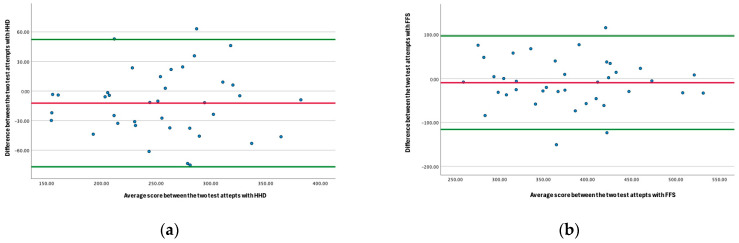
Bland–Altman plots demonstrating bias and 95% limits of agreement for the HHD (**a**) and FFS (**b**). The blue points represent the individual measurement differences; the green lines indicate the upper and lower 95% limits of agreement. Most values fell within the limits of agreement which demonstrated acceptable agreement and minimal systematic bias between the measurements.

Although the HHD demonstrated a statistically significant difference between the two testing occasions (mean change −12.27 N, *p* = 0.027), this represents within-device variability over time rather than a difference between devices. The FFS showed no significant change between occasions (mean change −9.41 N, *p* = 0.292). Importantly, both devices demonstrated a similar direction and magnitude of change across assessments. Bland–Altman analysis showed that the mean bias between the devices was small, with all values falling within the 95% limits of agreement. Linear regression analyses confirmed no proportional bias for either device (HHD β = 0.003, *p* = 0.987; FFS β = 0.057, *p* = 0.732), indicating that differences between the devices did not vary as a function of force magnitude. These results are demonstrated in Table 3 and visually represented in Figure 2. Collectively, the small mean changes observed for both devices and the absence of proportional bias support the construct validity of the HHD relative to the FFS in measuring isometric hip adduction strength.

## 4. Discussion

This study demonstrated that the HHD provides valid and reliable measurements of isometric hip adduction strength in elite youth football players. Therefore, HHDs may serve as a practical, portable, and cost-effective alternative to fixed dynamometry for field-based strength assessment, rehabilitation monitoring, and injury-prevention screening in elite football environments.

### 4.1. Intra-Rater Reliability

The present study demonstrated excellent intra-rater reliability for the HHD (ICC = 0.90) and good reliability for the FFS (ICC = 0.85) when measuring isometric hip adduction strength in elite academy footballers. These values are consistent with prior findings by Fulcher et al. (2010) [22] and Desmyttere et al. (2019) [6]. It is important to note that the HHD and FFS use different sensor technologies and data sampling frequencies, which may contribute to absolute value differences even when testing the same construct. Therefore, the FFS and HHD should not be used interchangeably for absolute values, but both machines can be utilised to track relative changes in the AST and monitor trends over time.

Thorborg et al. (2010) [20] emphasised the importance of external fixation when using HHDs in strong individuals to reduce tester-induced variability. In this study, a standardised squeeze position was used, placing the HHD between the knees in supine, allowing natural bilateral limb compression to stabilise the device. This reflects the approach used in real-world screening within elite football, offering strong ecological validity. Despite the lack of rigid external fixation, the HHD achieved comparable reliability to the fixed-frame FFS, suggesting that this natural stabilisation method provides sufficient mechanical consistency for reproducible measures. Supporting absolute reliability metrics reinforces these findings. SEM and MDC values were proportionally small relative to mean strength outputs (HHD: SEM = 12.3 N, MDC = 34.2 N; FFS: SEM = 21.5 N, MDC = 59.6 N), indicating that the devices produce consistent results over time. The MDC represents the smallest difference that can be interpreted as a true change in performance, beyond typical measurement error or day-to-day variability. Therefore, for practitioners monitoring an athlete’s progress, any change greater than the MDC (e.g., >34 N for HHD) should be interpreted as a meaningful physiological adaptation or decline in strength capacity, rather than random variation or tester inconsistency. As the MDC was lower using the HHD than the FFS, the findings support using the HHD for performance tracking.

Bland–Altman analysis revealed narrow limits of agreement (HHD: LOA = 11.75; FFS: LOA = 14.96) and no systematic bias, while CV values remained low (HHD: 5.5%; FFS: 6.0%), consistent with findings from Light and Thorborg (2016) [10] and O’Connor et al. (2022) [9]. Overall, these findings suggest both the HHS and the FFS can provide stable, reproducible measures of isometric hip adduction strength. Furthermore, comparable reliability results suggest that the field-relevant HHD setup with appropriate standardisation offers a viable and practical alternative to fixed dynamometry for performance screening and rehabilitation benchmarking.

### 4.2. Construct Validity

Overall, the findings of this study support the construct validity of the HHD when compared with the FFS for evaluating the AST.

While the two devices produced different absolute force values, this discrepancy is consistent with previous literature and expected due to inherent differences in device design. Dunne et al. (2022) [23] compared an HHD with a similar fixed-frame dynamometer to the FFS and reported consistently higher peak force values from the fixed device. Although the systems differ, both utilise bilateral load cells and rigid frame setups, which likely contribute to greater force outputs. Additionally, Light et al. (2016) [10] demonstrated that variations in lever arm length and joint position significantly influence torque generation during hip adduction testing, reinforcing that methodological setup plays a key role in determining absolute values across devices. Despite these differences in magnitude, both devices in the present study followed consistent participant strength rankings, and results fell within the limits of agreement. Therefore, the findings suggest the HHD can accurately detect relative differences in adductor strength. These findings align with previous studies, which demonstrated concurrent validity across measurements taken from adolescent athletes [9] and elite Australian footballers [14]. Similarly, Light and Thorborg [10] emphasised the importance of standardised joint positioning and test protocols in achieving valid comparisons between dynamometers.

Interestingly, the HHD demonstrated a statistically significant difference between the two testing occasions, which the FFS did not. However, all values fell within the limits of agreement, and linear regression demonstrated no proportional bias. Therefore, these changes may represent within-device variability, learning effects, or biological adaptation that have occurred over four weeks between testing sessions.

Unlike previous studies in sub-elite adult players [7,8,22], this study focused on elite youth footballers within an academy performance pathway. This context enhances the ecological validity of the findings and supports the use of the HHD as a feasible solution for field-based strength monitoring in high-performance settings. Given the logistical constraints in elite football environments, the ability of the HHD to provide valid, comparable data to that of a fixed-frame system offers valuable flexibility in both performance profiling and rehabilitation benchmarking.

### 4.3. Limitations

One limitation of this study is that it did not consider the potential effects of bilateral deficits. Bilateral deficits are a well-documented phenomenon where the total force generated is less than the sum of unilateral efforts [24], which may be more influenced by dynamometer setup and body positioning than neural mechanisms [6,25].

The participant cohort consisted of elite academy footballers grouped by age in accordance with the Elite Player Performance Plan pathway [16]. Specifically, 18 players (47%) were from the Youth Development Phase and 20 players (53%) from the Professional Development Phase. Based on bio-banding classifications, most participants (63%) would be considered adolescents (typically aged 14–17 years) [26]. This developmental stage may affect strength outputs and limits generalisability to adult or female populations with differing levels of physical maturity.

In addition, no screening questionnaire was used to capture participants’ past medical history or prior groin-related injuries. Unrecorded injury history may have influenced individual test performance, introducing unmeasured variability. Minor differences in device hardware and sampling frequency between the HHD and FFS may also have contributed to small variations in absolute peak-force outputs, although these did not affect the construct validity findings.

Finally, this study did not evaluate inter-rater reliability. Therefore, these results only apply to scenarios where the same assessor performs repeated measurements. Therefore, these results only apply to scenarios where the same assessor performs repeated measurements. Although previous research [11,14] has demonstrated strong inter-rater agreement using both HHD and FFS, in applied settings, inter-rater variability can be substantial and should be investigated in future research.

## 5. Conclusions

This study demonstrated that the HHD is a reliable and valid tool for assessing isometric hip adduction strength using the AST in elite youth football players. The HHD showed excellent intra-rater reliability and strong construct validity compared to the FFS, supporting its use as a practical, portable, and cost-effective alternative for field-based monitoring and rehabilitation. These findings provide practitioners with an accessible option for regular adductor strength assessment. Future research should explore inter-rater reliability, bilateral deficit effects, and broader applicability across sexes, age groups, and sporting populations.

## Figures and Tables

**Table 1:** Participant eligibility criteria.

Inclusion Criteria	Exclusion Criteria
Premier League Elite Football Academy players of Category 2 level, aged 15–18 years of age. Players who train at least six hours per week, and their team has at least one game per week. Players should be able to participate in all training and games without any modification, following the accumulation of the club’s ACWR	Players who are currently injured and not able to participate fully in training or games Any player whose accumulation of ACWR is lower than 0.8 due to lack of training for any reason. Players who report any pain during the testing procedures

**Table 2:** Intra-rater reliability and variables of HHD and FFS.

Intra-Rater Reliability	ICC (95% CI)	SEM	SEM%	MDC	MDC%	CV%	LOA
HHD	0.90 (0.82–0.95)	12.3	4.8%	34.2	13.6%	5.5%	11.75
FFS	0.85 (0.71–0.92)	21.5	5.6%	59.6	15.5%	6.0%	14.96

ICC: intraclass correlation coefficient; CI: confidence interval; SEM: standard error of measurement; MDC: minimal detectable change; CV: coefficient of variation; LOA: limits of agreement; HHD: data using hand-held dynamometer; FFS: data using ForceFrame System. SEM and MDC values are displayed in Newtons (N).

**Table 3:** Paired *t*-test and linear regression.

Paired Test	Mean	SD	T	Sig.
HHD	−12.27	32.92	−2.297	0.027
FFS	−9.41	54.26	−1.069	0.292
Linear regression		Beta	T	Sig
HHD		0.003	0.017	0.987
FFS		0.057	−0.344	0.732

HHD: tests with hand-held dynamometer; FFS: tests with ForceFrame System; SD: standard deviation; T: t-value; Sig: statistical significance. SD values displayed in Newtons (N).

## Data Availability

The data presented in this study are available on request from the corresponding author. The data are not publicly available due to participant confidentiality and institutional data protection policies.

## Supplementary Materials

The following supporting information can be downloaded at: https://www.mdpi.com/article/10.3390/sports14020053/s1, Table S1: GRRAS Checklist for Reporting Reliability and Agreement Studies; Table S2: Participants Characteristics; Table S3: Descriptive force outputs.

## References

[1] Ergun M., Denerel H.N., Sebik O., Ertat K.A. (2013). Injuries in elite youth football players: A prospective three-year study. Acta Orthop. Traumatol. Turc..

[2] Ekstrand J., Hägglund M., Waldén M. (2011). Epidemiology of muscle injuries in professional football (soccer). Am. J. Sports Med..

[3] Werner J., Hägglund M., Waldén M., Ekstrand J. (2019). Hip and groin timeloss injuries in professional football: A 15-year prospective analysis of 17,000 injuries in male professional footballers. Br. J. Sports Med..

[4] Pulici L., Certa D., Zago M., Volpi P., Esposito F. (2023). Injury burden in professional European football (soccer): Systematic review, meta-analysis, and economic considerations. Am. J. Sports Med..

[5] Schoffl J., Dooley K., Miller P., Miller J., Snodgrass S.J. (2021). Factors associated with hip and groin pain in elite youth football players: A cohort study. Sports Med. Open.

[6] Desmyttere G., Gaudet S., Begon M. (2019). Test–retest reliability of a hip strength assessment system in varsity soccer players. Phys. Ther. Sport.

[7] Holmich P., Thorborg K., Dehlendorff C., Krogsgaard K., Gluud C. (2014). Incidence and clinical presentation of groin injuries in sub-elite male soccer. Br. J. Sports Med..

[8] Engebretsen A.H., Myklebust G., Holme I., Engebretsen L., Bahr R. (2010). Intrinsic risk factors for groin injuries among male soccer players: A prospective cohort study. Am. J. Sports Med..

[9] O’Connor F., Falvey É., Comyns T., Richter C., King E. (2022). Reliability and validity of common hip adduction strength measures: The ForceFrame strength testing system versus the sphygmomanometer. Phys. Ther. Sport.

[10] Light N., Thorborg K. (2016). The precision and torque production of common hip adductor squeeze tests used in elite football. J. Sci. Med. Sport.

[11] Aerts F., Sheets H., Anderson C., Bussie N., Hoskins R., Maninga A., Novak E. (2025). Reliability and agreement of hand-held dynamometry using three standard rater test positions. Int. J. Sports Phys. Ther..

[12] Crow J.F., Pearce A.J., Veale J.P., VanderWesthuizen D., Coburn P.T., Pizzari T. (2010). Hip adductor muscle strength is reduced preceding and during the onset of groin pain in elite junior Australian football players. J. Sci. Med. Sport.

[13] Bahr R. (2016). Why screening tests to predict injury do not work—And probably never will. Br. J. Sports Med..

[14] Ryan S., Kempton T., Pacecca E., Coutts A.J. (2019). Measurement properties of an adductor strength-assessment system in professional Australian footballers. Int. J. Sports Physiol. Perform..

[15] Kottner J., Audige L., Brorson S., Donner A., Gajewski B.J., Hróbjartsson A., Roberts C., Shoukri M., Streiner D.L. (2011). Guidelines for reporting reliability and agreement studies (GRRAS). Int. J. Nurs. Stud..

[16] Premier League Elite Player Performance Plan (EPPP). https://www.premierleague.com/youth/EPPP.

[17] Gabbett T.J., Kennelly S., Sheehan J., Hawkins R., Milsom J., King E., Whiteley R., Ekstrand J. (2016). If overuse injury is a ‘training load error’, should undertraining be viewed the same way?. Br. J. Sports Med..

[18] Ishøi L., Petersen J., Thornton K., DeLang M. (2025). Maximal strength testing using the long-lever hip adduction squeeze test in youth elite football (soccer) players: A four-week reliability study. Int. J. Sports Phys. Ther..

[19] Anderson L., Orme P., Di Michele R., Close G.L., Morgans R., Drust B., Morton J.P. (2016). Quantification of training load during one-, two- and three-game week schedules in professional soccer players from the English Premier League. J. Sports Sci..

[20] Thorborg K., Petersen J., Magnusson S.P., Hölmich P. (2010). Clinical assessment of hip strength using a hand-held dynamometer is reliable. Scand. J. Med. Sci. Sports.

[21] Bartko J. (1966). The intraclass correlation coefficient as a measure of reliability. Psychol. Rep..

[22] Fulcher M.L., Hanna C.M., Elley C.R. (2010). Reliability of handheld dynamometry in assessment of hip strength in adult male football players. J. Sci. Med. Sport.

[23] Dunne C., Callaway A.J., Thurston J., Williams J.M. (2022). Validity, reliability, minimal detectable change, and methodological considerations for HHD and portable fixed frame isometric hip and groin strength testing: A comparison of unilateral and bilateral testing methods. Phys. Ther. Sport.

[24] Henry F.M., Smith L.E. (1961). Simultaneous vs. separate bilateral muscular contractions in relation to neural overflow theory and neuromoter specificity. Res. Quarterly. Am. Assoc. Health Phys. Educ. Recreat..

[25] Simoneau-Buessinger E., Leteneur S., Toumi A., Dessurne A., Gabrielli F., Barbier F., Jakobi J.M. (2015). Bilateral strength deficit is not neural in origin; rather due to dynamometer mechanical configuration. PLoS ONE.

[26] Lloyd R.S., Oliver J.L. (2012). Long-term athletic development—Part 1: A pathway for all youth 2015. Strength Cond. J..

