# Supplementary material for: Intra-Rater Reliability and Construct Validity of Hand-Held Dynamometry to Evaluate the Hip Adductor Squeeze Test in Elite Youth Football Players
Source: Sports (Basel). 2026 Feb 3;14(2):53. doi: 10.3390/sports14020053 (PMC12944607; doi:10.3390/sports14020053)
Supplement: Supplementary file 1 [file sports-14-00053-s001.zip › sports-4080443-supplementary/sports-4080443-supplementary.pdf]

Supplementary Material File S1

Table S1. GRRAS Checklist for Reporting Reliability and Agreement Studies

Section	Item	Checklist Item	Page in Manuscript
**Title & Abstract**	1	Identify in title or abstract that intrarater reliability or agreement was investigated	Title & Abstract (p. 1)
**Introduction**	2	Name and describe the diagnostic or measurement device of interest	p. 1
	3	Specify the subject population of interest	p. 2
	4	Specify the rater population of interest (if applicable)	p. 2-3
	5	Describe what is already known about reliability and agreement; provide rationale	p. 1-2
**Methods**	6	Explain how the sample size was chosen; state raters, subjects, replicates	p. 2-3
	7	Describe sampling method	p. 3
	8	Describe measurement process (intervals, blinding, clinical info)	p. 3-6
	9	State whether ratings were conducted independently	p. 2
	10	Describe the statistical analysis	p. 4
**Results**	11	State number of raters and subjects, and replicate observations	p. 2-3
	12	Describe rater and subject sample characteristics	p. 2-3
	13	Report estimates of reliability/agreement including statistical uncertainty	p. 5–7, Tables 2 & 3
**Discussion**	14	Discuss practical relevance of findings	p. 6–9
**Auxiliary Material**	15	Provide detailed results (e.g. online/supplementary)	Appendix / Supplementary Materials

Based on table I in: Kottner J, Audige L, Brorson S, et al. Guidelines for reporting reliability and agreement studies (GRRAS). Int J Nurs Stud 2011, 48(6):661–71.

Table S2. Participant characteristics

Descriptive demographic information for all players included in the study who completed both testing occasions.

Table S1. Participants descriptive data

**Number of participants**	38
Age Range (years)	16.4 ± 1.5
Height (cm)	181.7 ± 6.3
Body Mass (Kg)	74.4 ± 7.07
**Foot dominance**	
Right footed players	32
Left footed players	6
Under-16 players	18
Under-18 players	12
Under-21 players	8

[1] **Legend**: cm, centimeters: Kg, Kilograms

Table S3. Descriptive force outputs.

Table S2. Strength Scores Values recorded with each device on each test.

Test	Minimum	Maximum	Mean	SD
HHD1	139.5	377.83	249.94	58.94
HHD2	162.67	387.33	262.21	58.85
FFS1	255.17	525.25	373.86	73.56
FFS2	238.1	551.64	382.13	76.46

HHD1: First test with Hand-Held Dynamometer; HHD2: Second test with Hand- Held Dynamometer; FFS1: First test with ForceFrame System; FFS2: Second test with ForceFrame System; SD: Standard deviation. All values displayed at Newtons (N)
